# Teratoma of the lumbosacral region: a case report

**DOI:** 10.1186/1752-1947-5-370

**Published:** 2011-08-12

**Authors:** Mohd Faheem, Hasan H Syed, Dinesh Kardam, Veena Maheshwari, Roobina Khan, Atul Sharma

**Affiliations:** 1 Department of Surgery, Jawahar Lal Nehru Medical College, Aligarh Muslim University, Aligarh, India- PIN 202002; 2 Department of Pathology, Jawahar Lal Nehru Medical College, Aligarh Muslim University, Aligarh, India-PIN 202002

## Abstract

**Introduction:**

Teratoma is a tumor that usually arises from one or more germ layers. They are most commonly found in the sacrococcygeal region and have a female preponderance. We present a very rare case of a boy with a benign cystic teratoma in the lumbosacral region.

**Case presentation:**

A 16-year-old Indian boy presented to our hospital with a history of a lump in the lower back region since birth. Initially, it was small, but its size increased gradually over time to a size of 15 cm × 15 cm at presentation. There were no other associated abnormalities. Investigations revealed the lump to be a benign cystic teratoma. The patient underwent surgery, and the whole tumor, from its base to the vertebrae, was excised. Bisection of the tumor revealed that it contained hair and pultaceous material consistent with a teratoma, which was later confirmed by histopathologic examination.

**Conclusion:**

Benign cystic teratomas should be diagnosed and managed aggressively because they generally have a greater tendency to progress toward malignancy. After extensively searching the case report database, we arrived at the conclusion that this was a rare case of a benign cystic teratoma in the lumbosacral region in a boy.

## Introduction

Teratomas are germ cell tumors primarily composed of multiple types of cells derived from one or more of the three germ layers [1]. The term "teratoma," which literally means "monster" in Greek, was coined by Virchow. Teratomas can be categorized into two types: mature and immature. Mature teratomas can further be classified as solid or cystic (dermoid cysts). A dermoid cyst is lined with epithelium that contains tissues and cells normally present in the skin layer, including hair follicles and sebaceous and sweat glands. The most common locations are the sacrococcygeal region (57%), followed by the gonads (29%), the mediastinal region (7%), the retroperitoneum (3%), the cervical area, and the cranium [2-4]. The "sacrococcygeal" term is a misnomer because teratomas almost always arise from the coccyx and not from the sacral region. Teratomas show a female preponderance at a ratio of four to one [5,6]. However, the occurrence of a lumbosacral teratoma in a male patient is fairly rare. Hence, the present case report is intended to highlight this extremely rare occurrence regarding the tumor site.

## Case report

**Figure 1:**
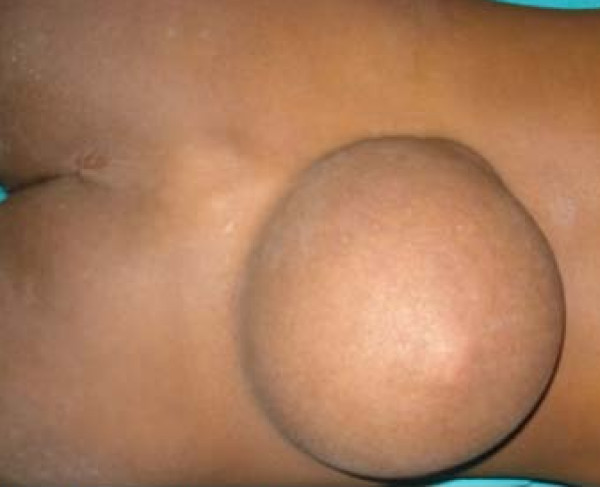
**Photograph showing the teratoma in the lumbosacral region**.

A 16-year-old Indian boy was brought to our hospital with swelling in the midline lower back that had been present since birth (Figure 1). The swelling had gradually increased to its size at presentation and was associated with mild physical discomfort. Apart from these findings, there was no significant history as far as the patient's swelling was concerned.

**Figure 2:**
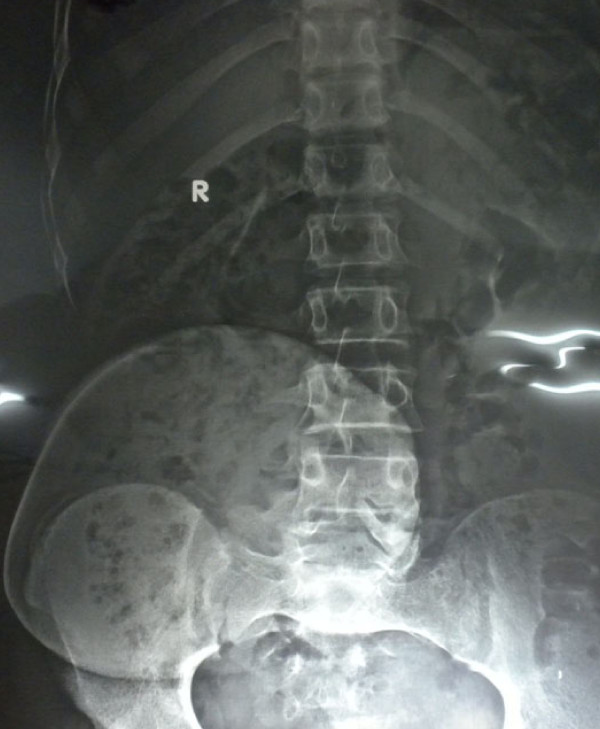
**Radiograph showing the well-defined outline of the teratoma**.

**Figure 3:**
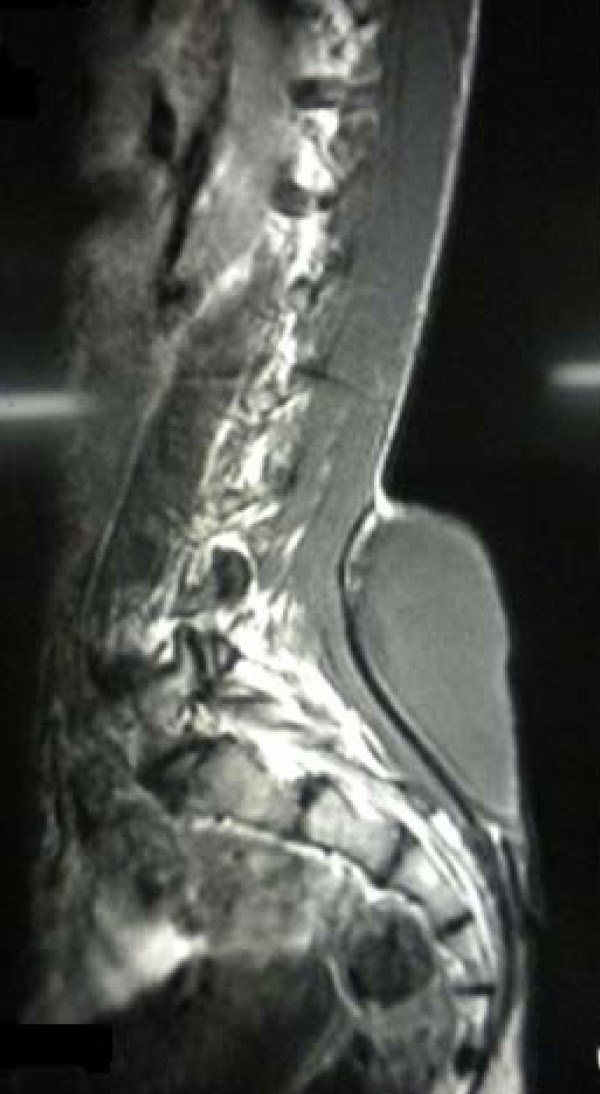
**MRI scan showing the teratoma at the level of the lumbosacral region**.

The initial examination revealed a cystic, non-mobile, non-tender mass approximately 15 cm × 15 cm in size attached to the back in the midline in the lumbosacral region. However, the patient's blood counts, urine analysis, and liver function test results were normal. Furthermore, the radiographs of the lumbosacral region showed a well-defined swelling 15 cm × 20 cm in size with a smooth margin from the L3 vertebra to the S3 vertebra (Figure 2). On the basis of our clinical suspicion of a cystic tumor, fine-needle aspiration cytology (FNAC) was performed to confirm the diagnosis. The results were positive for a mature cystic teratoma. Accordingly, the patient was prepared for surgery, and MRI was performed to establish the extent of the tumor. MRI of the lumbosacral spine revealed a well-defined lesion in the midline extending to the right gluteal region in the subcutaneous plane from approximately the L3-L4 to the S4 vertebrae and crossing the midline. It was further observed that the tumor was hyperintense on T1-weighted images and hypointense on T2-weighted images, which was suggestive of fat contents. There was no obvious communication with the spinal cord (Figure 3).

The tumor was excised by creating an elliptical incision over the cyst. A whitish yellow, well-encapsulated, non-mobile mass was observed. The tumor was carefully dissected to allow us to reach the base, which was found to be attached to the L5 lumbar vertebra. The attachments, along with a small piece of lumbar vertebra, were also removed to minimize the chance of recurrence.

## Discussion

A teratoma is an encapsulated tumor with components resembling normal derivatives of all three germ layers [2]. Teratomas usually arise as masses in the sacrococcygeal region [7]. Their predilection for this area is most likely related to the large number of pluripotent cells usually found in the caudal region of the embryo, which is closely associated with the distal sacrum and coccyx. Being encapsulated, teratomas are usually benign, although sometimes malignant transformation may occur, mainly into squamous cell carcinoma [1,8,9]. It is therefore recommended that they be excised as soon as possible. A mature teratoma is typically benign and is found more commonly in females, but immature teratomas are typically malignant and are found more often in males.

The other differential diagnoses considered in this case were lumbosacral lipomeningomyelocele, congenital lipoma, and sacrococcygeal teratoma. Lipomeningomyeloceles commonly occur in the lumbosacral area, but the MRI examination of our patient revealed no communication with the spinal cord, so this possibility was ruled out [10]. Similarly, congenital lipoma was also excluded from the differential diagnosis based on FNAC, which did not show any fat cells [11]. A sacrococcygeal teratoma almost always arises from the coccyx and not from the sacral area, so this possibility was ruled out on the basis of the findings suggested by the clinical examination and MRI [12].

The diagnosis of a teratoma is based mainly on histopathologic examination, although MRI is also helpful in determining its connection with the vertebral column or its extension into the spinal cord. Prenatally, teratomas are usually diagnosed on the basis of obstetric ultrasonography *in utero *[7]. They appear as a mixture of cystic and solid components. Recently, prenatal MRI has also been used in the imaging of antenatal fetal anomalies. Mothers carrying fetuses with cystic teratomas may develop polyhydramnios, which may lead to pre-term labor secondary to uterine distension. Volume reduction amniocentesis and tocolytics may be required to treat symptomatic polyhydramnios and prevent pre-term delivery [7]. In this case, the mother of the patient had not undergone any prenatal ultrasonography since she was illiterate and was not aware of the importance of prenatal ultrasonography in diagnosing neural tube defect in utero so she did not turn up for ultrasonography. She did not develop any difficulties during labor.

**Figure 4:**
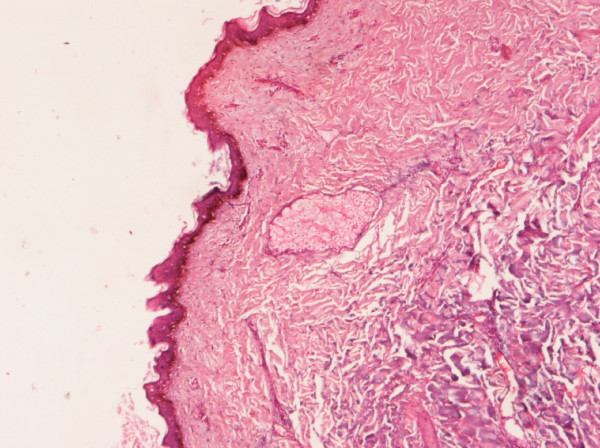
**Slide showing stratified squamous epithelium within the sebaceous gland**.

Evidence indicates that if the base is not excised along with its attachment to underlying bone, a teratoma may recur because it might contain totipotent cells. Therefore, complete excision is imperative [5,13]. However, in our patient, the base of the teratoma was found to be attached to the L5 vertebra, a small chip of which was removed along with its attachment. Furthermore, the excised specimen, which was sent for histopathologic examination, also revealed it to be a benign cystic teratoma (Figure 4).

The site of the teratoma in our patient was the L5 vertebra, which is extremely rare [14-17]. A study at the SMS Medical College, Jaipur, India, revealed only one case of this type of teratoma arising from the lumbosacral region (also in a female) among 75 cases of teratomas studied over a span of 22 years (Table 1) [13].

**Table 1:** Anatomic sites and sex distribution of teratomas^a^

Site	Patients, *n *(%)	Men, *n*	Women, *n*
Sacrococcygeal	49 (65.3)	12	37
Ovarian	10 (13.3)	-	10
Testicular	5 (6.7)	5	-
Oral cavity	3 (4.0)	1	2
Retroperitoneal	2 (2.7)	-	2
Cervical	2 (2.7)	2	-
Nasopharyngeal	1 (1.3)	-	1
Lumbosacral	1 (1.3)	-	1
Perineal	1 (1.3)	1	-
Gastric	1 (1.3)	1	-
Total	75	22 (29%)	53 (71%)

^a^Data are from [13].

## Conclusion

Teratomas are usually benign but sometimes may occur as malignant tumors. To avoid any diagnostic dilemma, it is significant to understand the rare presentation with regard to the tumor site and the possibility of malignancy. The case history and the very rare site of the tumor described in this report will help clinicians in diagnosing such cases and will help in enhancing clinical knowledge and experience for better treatment and patient care.

## Consent

Written informed consent was obtained from the patient for publication of this case report and any accompanying images. A copy of the written consent is available for review by the Editor-in-Chief of this journal.

## Competing interests

The authors declare that they have no competing interests.

## Authors' contributions

MF was a major contributor to the writing of the manuscript. HHS analyzed and interpreted the patient data. VM and RK performed the histologic examination. DK and AS helped in the writing of the manuscript. All authors read and approved the final manuscript.

## References

[B1] Kumar V Abbas AK Fausto N The Female Genital Tract Pathologic Basis of Disease 2006 7 St Louis: Elsevier 1099 1110

[B2] Teratoma, Cystic http://emedicine.medscape.com/article/281850-overview

[B3] Barksdale EM Jr Obokhare L Teratomas in infants and children Curr Opin Pediatr 2009 21 344 349 10.1097/MOP.0b013e32832b41ee 19417664

[B4] Azizkhan R Caty MG Teratomas in children Curr Opin Pediatr 1996 8 287 292 10.1097/00008480-199606000-00018 8814409

[B5] Legbo JN Opara WE Legbo JF Mature sacrococcygeal teratoma: case report Afr Health Sci 2008 8 54 57 19357734 PMC2408549

[B6] Sacrococcygeal Teratoma http://hcp.obgyn.net/ultrasound/content/article/1760982/1906534

[B7] Krishan S Solanki R Sethi SK Sacrococcygeal teratoma: role of ultrasound in antenatal diagnosis and management JHK Coll Radiol 2004 7 35 39

[B8] Shanbhogue LKR Bianchi A Doig CM Gough DCS Management of benign sacrococcygeal teratoma: reducing mortality and morbidity Pediatr Surg Int 1990 5 41 44

[B9] Terenziani M D'Angelo P Bisogno G Boldrini R Cecchetto G Collini P Conte M De Laurentis T Ilari I Indolfi P Inserra A Pira L Siracusa F Spreafico F Tamaro P Lo Curto M Teratoma with a malignant somatic component in pediatric patients: the Associazione Italiana Ematologia Oncologia Pediatrica (AIEOP) experience Pediatr Blood Cancer 2010 54 532 537 20049928 10.1002/pbc.22397

[B10] AANS Tethered Spinal Cord Syndrome http://www.aans.org/Patient%20Information/Conditions%20and%20Treatments/Tethered%20Spinal%20Cord%20Syndrome.aspx

[B11] Pierre-Kahn A Zerah M Renier D Cinalli G Sainte-Rose C Lellouch-Tubiana A Brunelle F Le Merrer M Giudicelli Y Pichon J Kleinknecht B Nataf F Congenital lumbosacral lipomas Childs Nerv Syst 1997 13 298 335 10.1007/s003810050090 9272285

[B12] Mahour GH Sacrococcygeal teratomas CA Cancer J Clin 1988 38 362 367 10.3322/canjclin.38.6.362 3141009

[B13] Sharma AK Sharma CS Gupta AK Sarin YK Agarwal LD Zaffar M Teratoma in pediatric age group: experience with 75 cases Indian Pediatr 1993 30 689 694 8282403

[B14] Reid SA Mickle JP Myelomeningocele occurring within a lumbosacral teratoma: case report Neurosurgery 1985 17 338 340 10.1227/00006123-198508000-00019 4033887

[B15] Bucy PC Haymond HE Lumbosacral teratoma associated with spina bifida occulta: report of a case with review of the literature Am J Pathol 1932 8 339 346 19970023 PMC2062683

[B16] Sharma MC Jain D Sarkar C Bhatnagar V Rishi A Suri V Garg A Lumbosacral Wilms' tumor as a component of immature teratoma associated with spinal dysraphism: a rare case and short literature review Fetal Pediatr Pathol 2009 28 201 208 19842874 10.3109/15513810903070654

[B17] Ibrahim AE Myles L Lang DA Ellison DW Case of the month: June 1998. 2 year old boy with lumbosacral mass Brain Pathol 1998 8 817 818 9804389

